# Clinical Application of *TERT* Promoter Mutations in Urothelial Carcinoma

**DOI:** 10.3389/fonc.2021.705440

**Published:** 2021-07-29

**Authors:** Yujiro Hayashi, Kazutoshi Fujita, George J. Netto, Norio Nonomura

**Affiliations:** ^1^Department of Urology, Osaka University Graduate School of Medicine, Suita, Japan; ^2^Department of Urology, Osaka General Medical Center, Osaka, Japan; ^3^Department of Urology, Kindai University Faculty of Medicine, Osakasayama, Japan; ^4^Department of Pathology, University of Alabama at Birmingham, Birmingham, AL, United States

**Keywords:** *TERT* promoter, telomere, urothelial carcinoma, bladder cancer, upper tract urothelial carcinoma, tumorigenesis, liquid biopsy, cell-free DNA

## Abstract

Urothelial carcinoma (UC) is a common urological malignancy with a high rate of disease recurrence. Telomerase activity, a hallmark of cancer characterized by overcoming the replicative senescence, is upregulated in over 90% of patients with UC. Somatic mutations in the promoter region of telomerase reverse transcriptase (TERT) are frequently detected in UC, and drive telomerase activity. Recent studies have demonstrated a strong association between *TERT* promoter mutation and tumorigenesis of UC. Also, *TERT* promoter mutation has great potential for diagnosis, as well as prognosis in UC treatment, and this is also applicable for the liquid biopsy techniques. In this review, we discuss the progress in these areas and highlight the challenges, clinical potential, and future direction for developing UC treatment methods.

## Introduction

Approximately 95% of cases of urothelial carcinoma (UC) are reported in the bladder (UBC: urothelial bladder carcinoma), and upper tract urothelial carcinoma (UTUC) that occurs in the renal pelvis or ureter corresponds to a small subset of UC (1). UBC is a globally common disease, with the incidence of 570,000 cases and 210,000 deaths reported in 2020 worldwide (2), and cigarette smoking is thought to be the most important risk factor for UC formation (3). Additionally, the exposure to aristolochic acid increased the risk of developing UTUC (4). Comprehensive tumor genome analysis provides a deeper understanding of the molecular biology of both UBC (5) and UTUC (6). Although there are some differences between UTUC and UBC in terms of embryological aspects and their association with the environmental exposure, these two types of carcinoma exhibit a close relationship in clinical practice, as they share some of the pathological and mutational signatures (7). In addition to *FGFR3* or *TP53* mutations, the promoter of telomerase reverse transcriptase (TERT) is among the most frequently mutated genomic region in UC tissue (8, 9). Because of the high recurrence rate and the need for definitive diagnosis, many patients with UBC and UTUC are often required to perform periodic invasive endoscopy tests, such as cystoscopy or ureteroscopy (10, 11). Although there are few reliable biomarkers of UC, liquid biopsy technique targeting the frequently mutated genes in UC tissue would help resolve this issue in a non-invasive manner. The growing evidences suggest that *TERT* promoter mutation, telomere length, and telomerase activity play crucial roles in UC tumorigenesis through inducing genomic instability (12–14), and act as clinically useful biomarkers tested using liquid biopsy technique (15). In this review, we summarize the current biological insights on *TERT* promoter mutation and telomerase activity in UC, and their clinical application in patients with UC.

## Role of Telomeres in Cancer

Human telomeres existing at the chromosomal ends play an essential role in protecting the chromosomes from being mistaken as the sites of DNA damage (15). Human telomeres are composed of a long double-stranded “TTAGGG” canonical repetitive sequence comprising thousands of bases (16). The telomeric ends are concealed and protected from DNA damage response through forming a T-loop structure. Telomeres are shortened by less than 50 base pairs in each cell division, which is attributed to the inability of DNA polymerase to duplicate the ends of DNA molecules. This issue of end-replication, and short dysfunctional telomeres eventually result in cell cycle arrest or senescence once the replication limit of cell division, known as the Hayflick limit, is reached (17). Therefore, telomere shortening-induced cellular senescence acts as a strong barrier against tumorigenesis. However, in cancer cells or cancer progenitor cells that acquire the ability to escape the cell cycle arrest pathway or cellular senescence, dysfunctional telomeres act as an origin of genomic instability, and accumulation of somatic mutations in a state referred to as telomere crisis (13). Meeker et al. demonstrated that telomere shortening was widespread in various cancer types using direct telomere-fluorescence *in situ* hybridization technique, whereas telomere length variability was prevalent in bladder cells with either short or long telomeres (18).

## Telomerase and *Tert*

In 1985, terminal telomere transferase, later referred to as telomerase, was first discovered by Carol Greider and Elizabeth Blackburn (19). They reported the enzymatic activity in *T. thermophila* extracts that synthesized and elongated telomeres for the first time, and were awarded the Nobel Prize in Physiology or Medicine in 2009. Later, telomerase activity was discovered in a number of species, including humans (16). Telomerase is a telomere-specific ribonucleoprotein polymerase composed of reverse transcriptase (TERT) and telomerase RNA component (TERC). TERC contains a CCCUAA telomere template in its core domain (20). TERT synthesizes TTAGGG telomeric repeats and elongates telomeres where TERC binds to the 3’ end of telomere (16). Telomerase activity was downregulated through silencing *TERT* in most human adult non-germline tissues, except for a few stem or progenitor cells. The absence of telomerase activity leads to cellular senescence in normal cells during aging. Conversely, almost all human cancer cells maintain telomere length over many cell divisions through activating either DNA recombination alternative lengthening of telomeres (ALT) or upregulating *TERT (* 16). Although the ALT pathway occurs in only about 10% to 15% of cancers, about 85% to 90% of all human cancers exhibit high rate of telomerase activation (16). For these reasons, the expression of telomerase or *TERT* is considered as a hallmark of cancer, which enables replicative immortality (16, 20).

## Two Hotspot Mutations in *Tert* Promoter Region

**Figure 1:**
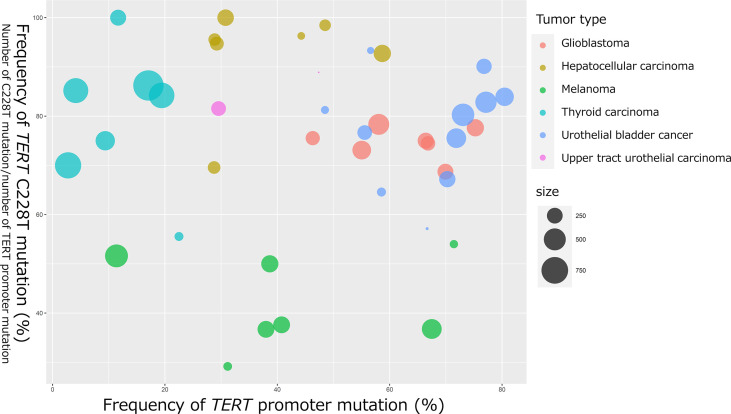
Bubble chart indicating the frequency of *TERT* promoter mutation and *TERT* C228T mutation in glioblastoma, HCC, melanoma, papillary thyroid cancer, UBC, and UTUC. The horizontal axis represents the frequency of *TERT* promoter mutations (number of *TERT* promoter mutations per number of all cases). The vertical axis represents the frequency of *TERT* C228T mutation (number of *TERT* C228T mutations per number of *TERT* promoter mutations). The bubble size represents the number of samples analyzed.

**Figure 2:**
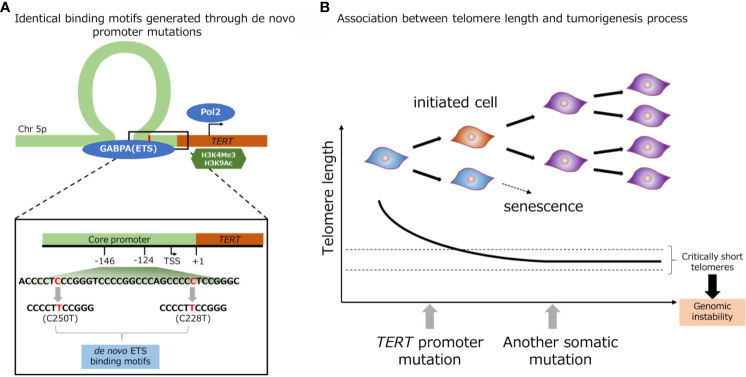
Identical *de novo* binding motifs generated through promoter mutations **(A)**. Schematic representation of TERT promoter mutation. Association between telomere length and tumorigenesis process **(B)**.

In 2013, two cancer-specific hotspot somatic mutations in *TERT* promoter that activate *TERT* transcription were first identified in melanoma (21, 22). Thereafter, *TERT* promoter mutations were found to be one of the most frequently mutated genomic regions in several tumor tissues (23). These mutations are located 124 and 146 bp upstream of the translation start site (TSS) of *TERT* gene and are referred to as C228T (g.1295228 C>T in GRCh37), and C250T (g.1295250 C>T in GRCh37). These two hotspot C to T transitions are typically heterozygous and form the same 11 base pair sequences of “CCCCTTCCGGG” (20) (**Figure 2A**). Interestingly, *TERT* promoter mutations are frequently detected in tumors originating from normal cells with low rates of self-renewal, such as glioblastoma (83%), melanoma (71%), UBC (67%), UTUC (47%), and hepatocellular carcinoma (HCC) (44%) (23). Conversely, some of the prevalent malignant tumors, including breast cancer, prostate cancer, thyroid cancer, colon cancer, stomach cancer, and leukemia, exhibit occasional *TERT* promoter mutations (23). Although the C228T and C250T hotspot mutations generate identical binding motifs for the transcription factors, there seems to be differences between these two mutations. We selected previous reports with a large number of cases or with seminal initial significance and analyzed the association of frequency of *TERT* C228T mutation in entire *TERT* promoter mutation and that of *TERT* promoter mutation in various types of tumor specimens [melanoma (21, 22, 24–28), glioblastoma (29–35), HCC (23, 36–41), papillary thyroid carcinoma (42–48), UBC (23, 49–58), and UTUC (23, 59)] (**Figure 1**). The size of the circles indicated the number of samples analyzed in each study. These data have demonstrated that *TERT* C228T mutation is frequently detected in multiple types of malignant tumors, including glioblastoma, hepatocellular carcinoma, thyroid carcinoma, and UC compared with C250T *TERT* mutation, except for skin melanoma (**Figure 1**). UC and glioblastoma exhibit a high frequency of *TERT* C228T mutation. Conversely, the ratio of *TERT* C228T mutation per *TERT* promoter mutations was reported to be high in thyroid cancer, but the frequency of *TERT* promoter mutations was lower than that in other types of cancer (**Figure 1**). The frequency of *TERT* promoter mutation in melanoma varied widely among several reports, and the ratio of *TERT* C228T mutation was lower than that in other types of cancer (**Figure 1**). In addition to the difference in this frequency, Li et al. demonstrated that to upregulate *TERT* or telomerase expression, it is necessary for tumor with C250T mutation, unlike C228T mutation, to introduce ETS1/2 transcription factor in cooperation with p52 downstream of the non-canonical NF-κB signaling pathway (60). Conversely, although the detailed biological mechanism of C228T mutation remains unclear, several reports suggest that C228T mutation acts more aggressively for a malignant phenotype than C250T mutation. In an experiment using genetically engineered hESCs, among a few types of *TERT* promoter mutations, only C228T mutation increased *TERT* mRNA expression, but not C250T or other types of mutation (61). Borah et al. demonstrated that *TERT* promoter mutation, mainly C228T, was associated with high levels of *TERT* mRNA expression, TERT protein expression, telomerase activity, and telomere length in bladder cancer cell lines (12). Furthermore, we found that C228T mutation detected in urinary cell-free DNA (cfDNA) was associated with bladder tumor recurrence in patients after transurethral surgery for NMIBC or radical nephroureterectomy for localized UTUC (57, 62). We also reported that *TERT* C228T mutation, but not C250T mutation, in the non-malignant urothelium of patients with NMIBC, was significantly associated with intravesical recurrence after transurethral surgery (58). As mentioned above, *TERT* C228T mutation was associated with worse clinical outcomes in some tumor types than *TERT* C250T mutation. These biological differences between the two hotspot mutations may be simply attributed to the difference in frequency, or due to differences in the transcription factor accessibility or epigenetic state; however, further investigation is required to be performed in the future.

## Potential Mechanism of *Tert* Promoter Mutation-Induced Tumorigenesis

Promoter mutations of *TERT* gene (C228T and C250T) creates a *de novo* binding site of GA-binding protein A (GABPA), an E-twenty-six (ETS) family transcription factor, in close proximity to a native ETS binding site in the wild-type *TERT* promoter (**Figure 2A**) (63). Akincilar et al. reported that *TERT* promoter mutation upregulates *TERT* expression *via* long-range chromatin interactions, acquisition of active histone methylation (H3K4Me3) and acetylation (H3K9Ac), and promotes GABPA binding to this region (**Figure 2A**) (64). Chiba et al. conducted a long-term experiment using cultured cells to determine the role of *TERT* promoter mutations in telomere maintenance during tumorigenesis (**Figure 2B**) (13). The authors showed that *TERT* promoter contributes in tumorigenesis in two steps: immortalization of cells and promotion of genomic instability. In the first step, the point mutations in the promoter region of *TERT* gene do not prevent the bulk telomere shortening, but preferentially act on critically short telomeres, thereby delaying the replicative senescence and extending the growth limit through maintaining the length of telomere. In the second step, the genomic instability is increased through the fusion of extremely short telomeres, and at the same time, telomerase expression is gradually increased, ensuring further proliferation for cell immortalization (13). A number of studies have showed evidences that *TERT* promoter mutation, coupled with telomerase activation were associated with early genetic event during tumorigenesis. The *TERT* promoter mutation was identified in benign follicular thyroid adenoma (65), hepatocellular adenoma (66), benign nevi (67), as well as non-malignant urothelium (58). Furthermore, benign follicular thyroid adenoma with *TERT* promoter mutation express *TERT* mRNA and telomerase activity. Taking the fact that *TERT* promoter mutation was detected in various types of pre-cancerous lesion including benign lesions, and the fact that genomic instability was induced by *TERT* promoter mutation into consideration, *TERT* promoter mutation thought to associated with various type of tumorigenesis.

## *Tert* Promoter Mutation in UC and Tumorigenesis

Based on histopathological and molecular observations, there is potential multistep model in UC tumorigenesis. In UC tissue, *TERT* promoter mutations are frequently detected in pre-cancerous lesions and high grade/stage UBC, or rare variant pathologies with an aggressive phenotype (**Table 1**) (58, 68–73). Although *TERT* promoter mutations were detected in the pathologically normal urothelium of patients with NMIBC (58), no mutation was detected in the benign proliferative bladder urothelium, including cystitis, nephrogenic adenoma, or inverted papilloma (74). These results indicate that *TERT* promoter mutation might occur during the trunk event of cancer clonal evolution and is thought to play a crucial role in UBC tumorigenesis. However, the collection of longitudinal samples from a single patient with malignant tumor over a long period of tumorigenesis poses a major challenge in studying tumor evolution. Therefore, mathematical algorithmic methods to infer tumorigenesis using cross-sectional data of tumor specimens from multiple patients have been proposed to solve this problem. We have demonstrated that this theory of tumorigenesis with *TERT* promoter as the axis of genome evolution of UBC is supported by phylogenetic inference using CAPRI model based on custom heuristic optimization of cross-sectional genomic data of tumor specimens from multiple patients with UBC (75). Although the biological mechanism of *TERT* promoter mutation-induced tumorigenesis is still unknown, these data support that genomic instability caused by *TERT* promoter mutations are essential for bladder cells with a low rate of self-renewal to acquire multiple somatic mutations and undergo malignant transformation in multistep tumor formation in UC.

**Table 1:** Frequency of *TERT* promoter mutation in different types of bladder tumor, including those with rare variant histology.

	Frequency of *TERT* promoter mutation (%)
PUNLMP (69)	63 (19/30)
Urothelial carcinoma (58)	52 (28/53)
Micropapillary variant (70)	100 (33/33)
Adenocarcinoma (71)	57 (4/7)
Squamous cell carcinoma (72)	80 (12/15)
Small cell carcinoma (73)	100 (11/11)
Plasmacytoid variant (74)	60 (6/10)

## Liquid Biopsy

Liquid biopsy allows the analysis in a less invasive, rapid, and sequential manner, and also to overcome the heterogeneity of tumors. Conventional next generation sequencing encountered sequencing error and cannot detect the mutation less than 5% due to error during PCR or reading process. By adding unique molecular identifiers, SafeSeqs technology could overcome these problems, enabling to distinguish between errors and “true mutations” by analyzing each read for each unique molecular identifier (76). This SafeSeqs technology can offer high sensitivity with a mutant allele frequency of 0.05%. droplet digital PCR can also offer highly sensitive analysis for molecular analysis. Droplet digital PCR technology can detect mutant alleles as few as 0.01% by analyzing ~20,000 of partitioned droplets using microfluidics technology (77). These technological advances in unique molecular identifiers, droplet digital PCR, and bioinformatics have made it possible to detect genetic mutations with greater sensitivity in body fluids.

Therefore, the genetic analysis using these techniques can provide useful molecular information and offer various advantages not only as a non-invasive biomarker, but also as an early indicator of minimal residual disease, recurrence, drug resistance, or metastasis. Therefore, it is believed to replace the tissue needle biopsy technique in the near future (15). Liquid biopsy is indispensable for the realization of precision medicine, as the target samples, including plasma cell-free DNA (cfDNA), urinary cfDNA, or urine pellet DNA are frequently analyzed in the UC field. Plasma cfDNA is released into the blood from both cancerous and non-cancerous tissues. The usefulness of tumor-derived cfDNA in plasma in various types of advanced carcinomas has been widely reported as a biomarker, but there is little evidence for the usefulness and efficacy of plasma cfDNA in localized cancer because the absolute amount of circulating tumor-derived cfDNA is low. Conversely, for the patients with UC, tumor-derived DNA can be detected in urine even in localized, tiny, or early-stage carcinoma, since unlike other carcinomas, UC is in constant contact with the urine (78, 79). Therefore, urinary DNA (urine pellet DNA and urinary cfDNA) can provide useful genetic analysis as a non-invasive tool for UC. There is growing evidence regarding the clinical application of liquid biopsy analysis for the patients with UC. Although there are few reports examining only *TERT* promoter mutations as target genomic region, *TERT* promoter mutations are frequently used in combination with other oncogenic mutations as a comprehensive panel analysis in liquid biopsy for the patients with UC, because of their high mutation frequency (76). UROSEEK has shown clinical potential for the detection of UC, which detects mutations in *TERT* promoter combined with *FGFR3, TP53, CDKN2A, ERBB2, HRAS, KRAS, PIK3CA, MET, VHL*, and *MLL* (8). We demonstrated clinical potential of *TERT* promoter in combination with *FGFR3* mutation as a simple droplet digital PCR assay (57, 62). Since the promoter region of *TERT* gene has a high GC content (over 80%), amplification of this region using PCR is more challenging than that of the genomic regions in the exome. To amplify these regions efficiently, some additives are used to prevent non-specific amplification of products during PCR.

## Clinical Potential of *Tert* Promoter Mutations in Urine Liquid Biopsy for UC

**Figure 3:**
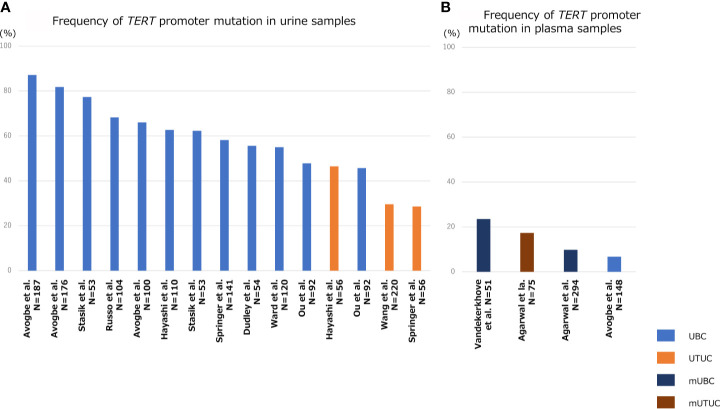
Bar diagram indicating the frequency *TERT* promoter mutation in liquid biopsy specimens of patients with UBC, UTUC, mUBC (metastatic UBC), and mUTUC (metastatic UTUC). Frequency of TERT promoter mutation in urine samples **(A)**, and that in plasma samples **(B)**.

**Figure 3** shows the association of frequency of *TERT* promoter mutation in plasma or urine specimens from patients with UTUC or UBC. The sensitivity of *TERT* promoter mutation assessed using urine liquid biopsy was relatively high, ranging from approximately 50% to 70% for UBC and 30–50% for UTUC (**Figure 3A**) (8, 57, 62, 80–87), which was consistent with the frequency of *TERT* promoter mutations in UC tissues (**Figure 1**). Springer et al. demonstrated the clinical application of urinary pellet analysis using targeted sequence panel “UROSEEK”, including *TERT* promoter mutations in the patients with UC (8). Several researchers reported that the overall concordance in *TERT* promoter mutation between urine and tumor tissue was high in both UBC and UTUC (92%, cfDNA in UBC; 89–94%, urine pellet in UTUC; 73–90%, urine pellet in UBC) (83), suggesting that urine liquid biopsy is sufficient for analyzing *TERT* promoter mutational status in both UBC and UTUC, and exhibits clinical potential for UC diagnosis. Several studies have shown that *TERT* promoter mutations, mainly C228T, were detectable up to 10 years prior to UBC formation in urine liquid biopsy of patients without visible tumor (86). Additionally, we reported that *TERT* C228T mutation in urinary cfDNA was associated with bladder tumor recurrence after transurethral surgery for NMIBC or radical nephroureterectomy for UTUC (57, 62). For these reasons, the detection of *TERT* C228T mutation in urine samples may act as a potential prognostic factor, as well as a diagnostic biomarker, in the clinical setting for UC treatment.

## Clinical Potential of *Tert* Promoter Mutations in Plasma Liquid Biopsy for Advanced UC

Since the quantity of plasma cfDNA is low in localized or non-muscle invasive tumors, it is difficult to detect tumor-derived mutations in blood samples from patients with early-stage UC. Therefore, the majority of previous studies on plasma cfDNA have focused mainly on metastatic UC. The positive rate of *TERT* promoter mutation in plasma cfDNA ranged from 10–23% in metastatic UBC and 17% in metastatic UTUC (**Figure 3B**) (87–89). Avogbe et al. reported that *TERT* promoter mutation was detected in 6.7% of plasma samples from patients with localized UBC (**Figure 3B**) (87).

## Challenges and Future Directions of Tert as a Potential Therapeutic Target

Researchers are now paying more attention to the regulation of telomerase or *TERT* because of their unique characteristic of low or no expression in normal somatic cells, but high expression in cancer cells. Many studies have been conducted on *TERT*-regulating factors, such as epigenetic modifications or telomerase inhibitors. With regard to methylation, there are two regions of *TERT* promoter: the unmethylated proximal *TERT* core promoter, and the hypermethylated region called THOR the *TERT* hypermethylated oncologic region (THOR) located at the distal region of the promoter. This interesting distinct methylation pattern indicates that the core promoter region and THOR are functionally different loci regulating *TERT* (90). Co-occurrence of THOR methylation and *TERT* promoter mutation was associated with a high risk of disease recurrence and progression in UBC (54). Although the exact biological mechanism is still unclear, THOR methylation might play an important role in regulating *TERT* expression and may be a potential target for cancer treatment.

A few telomerase peptide vaccines based on a novel antigen generated by T cells have been used in clinical trials for various types of cancers. In a phase I/II study, INO-5401 plus INO-9012 was administered to the patients with advanced or metastatic UC in combination with atezolizumab (NCT03502785). IN-5401 and INO-9012 are both mixtures of synthetic plasmids, including TERT antigen. Peptide vaccines have been used in various clinical trials with limited success. Since various reports indicate that *TERT* promoter mutations occur at an early stage of tumorigenesis in UC, these telomerase-targeted therapies might be effective for early-stage UC or prevention of UC recurrence, but not for advanced UC.

## Conclusion

The biological function of *TERT* promoter mutations in UC is gradually being well defined. There are growing evidences that *TERT* promoter mutation followed by genomic instability is a key step in the tumorigenesis of UC. Furthermore, the detection of *TERT* promoter mutations in bodily fluids using liquid biopsy is a potential biomarker for UC diagnosis as well as prognosis. Although larger prospective studies are required, liquid biopsy targeting *TERT* promoter mutation might make a significant contribution to improve the prognosis and quality of life of patients with UC in the near future.

## Author Contributions

Conception and design: all authors. Administrative support: KF. Provision of study materials or patients: all authors. Collection and assembly of data: YH. Data analysis and interpretation: YH. Manuscript writing: YH. All authors contributed to the article and approved the submitted version.

## Funding

This study was supported by Osaka University Grant, and JSPS KAKENHI Grant Number JP 20K18139.

## Conflict of Interest

GN: Equity or royalty from the licensed technologies from Johns Hopkins that are related to the work described in this paper.

The remaining authors declare that the research was conducted in the absence of any commercial or financial relationships that could be construed as a potential conflict of interest.

## Publisher’s Note

All claims expressed in this article are solely those of the authors and do not necessarily represent those of their affiliated organizations, or those of the publisher, the editors and the reviewers. Any product that may be evaluated in this article, or claim that may be made by its manufacturer, is not guaranteed or endorsed by the publisher.

## Abbreviations

cfDNA, cell-free DNA; HCC, hepatocellular carcinoma; NMIBC, non-muscle invasive bladder cancer; TERC, telomerase RNA component; TERT, telomerase reverse transcriptase; UBC, urothelial bladder cancer; UC, urothelial carcinoma; UTUC, upper tract urothelial carcinoma.
